# Role of Sensory Stimulation in Amelioration of Obstructive Sleep Apnea

**DOI:** 10.1155/2011/596879

**Published:** 2011-04-05

**Authors:** Mak Adam Daulatzai

**Affiliations:** Sleep Disorders Group, EEE, Melbourne School of Engineering, The University of Melbourne, ICT Building, 3rd Floor, Room no. 344, 111 Barry Street, Parkville, VIC 3010, Australia

## Abstract

Obstructive sleep apnea (OSA), characterized by recurrent upper airway (UA) collapse during sleep, is associated with significant morbidity and disorders. Polysomnogram is employed in the evaluation of OSA and apnea-hypopnea number per hour reflects severity. For normal breathing, it is essential that the collapsible UA is patent. However, obstruction of the UA is quite common in adults and infants. Normally, important reflex mechanisms defend against the UA collapse. The muscle activity of UA dilators, including the genioglossus, tensor palatini (TP), and pharyngeal constrictors, is due to the integrated mechanism of afferent sensory input → to motor function. Snoring is harsh breathing to prevent UA obstruction. Unfortunately, snoring vibrations, pharyngeal suction collapse, negative pressure, and hypoxia cause pathological perturbations including dysfunctional UA afferent sensory activity. The current paper posits that peripheral sensory stimulation paradigm, which has been shown to be efficacious in improving several neurological conditions, could be an important therapeutic strategy in OSA also.

## 1. Introduction

Obstructive sleep apnea (OSA) is characterized by recurrent upper airway (UA) obstruction, hypoventilation, hypoxia, and hypoxemia. Two cardinal features of OSA are (1) decrease in UA dilator muscles' force during sleep and (2) narrowed cross-sectional pharyngeal lumen that presents a significant mechanical constraint to normal ventilation. During UA collapse, ventilation is compromised (hypopnea) or absent (apnea), and hypoxia and hypercapnia develop. These blood gas changes increase respiratory drive until the UA reopens, at which time ventilation increases to reverse the blood gas abnormalities. This scenario reflects a dysfunctional genioglossus (a major UA dilator muscle) activity during sleep, in conjunction with a hypotonic pharynx that collapses. 

OSA affects *∼*4% of men and *∼*2% of women and is strongly linked to obesity. Owing to recurrent UA obstruction and hypoventilation, clinical consequences due to OSA (in most if not all sufferers) are several, ranging from—cardiovascular disease (hypertension, myocardial infarction, heart failure), stroke, metabolic derangement, respiratory failure, and cognitive dysfunction (see [1, 2]). Recent Australian and American studies have documented OSA to be an important risk factor from all-cause death-being as high as 33% in patients with severe OSA. The untreated OSA patients are at even greater risk, being 3.8 times more likely to die from any cause and 5.2 times more likely to die from cardiovascular conditions. Therefore, a thorough understanding of the pathogenesis of OSA, and effective therapeutic strategies, are essential to combat this important public health problem.

Moving a muscle involves neuromuscular communication. The response to contract by a muscle originates with sensory activation-simply put as in stepping on sharp object or touching a hot cup. The brain then sends a message to the muscle to respond. This pathway involves the afferent sensory limb to the brain and the motor nerve limb to the muscle. This well-characterized sensory-motor reflex pathway for muscle contraction is universal-operating in normal UA dilator musculature as well; however, this particular bidirectional loop is dysfunctional in OSA. Consequently, the compromised afferent input in conjunction with dysfunctional motor activity of dilator muscles influence ventilation causing hypopnea/apnea. Considerable research has focused on issues pertaining to sensory-motor integration [3]. Recent studies have shown how sensory information and premotor networks control the motor output. Sensory transmission combined with motor output, plus inhibitory synaptic transmission from interneurons of premotor networks, modulate this input → output pathway. These mechanisms play an important role in adapting the operation of central networks to external demands and thus help optimize sensory-motor integration [4]. Using a variety of available simple noninvasive, nonpharmacological procedures, one can stimulate UA sensory receptors of disparate modalities, and hence their interconnected efferent motoneurons, to activate the UA dilator muscles. Part of the current work has been published as an abstract [5]. 

## 2. Pharynx and Genioglossus: General

Pharynx and genioglossus are striated muscles; they undergo atrophy and degeneration when subjected to disuse and diseases. They are not only motor organs, but also the regions where a variety of sensory receptors are located. The sensory receptors are located in the mucosa which lines the genioglossus and the UA. The mucosal Proprioceptors transmit touch, temperature, pressure, and pain sensations. Sensory responses are also elicited by air stimuli; the airflow receptors respond to the air puff and cold or warm air humidified by the nose. The sensory receptors respond to the respiratory movements and changes in size and volume as well. Thus any stretch or mechanical alterations would provide important sensory feedback; the perception and response to the above stimuli by the CNS conceivably provide required motor efferent response, essential for various appropriate UA neurophysiological functions. The tactile and chemoreceptors, though not very active during food propulsion, profoundly affect ventilation when stimulated. These receptors provide dynamic sensory feedback. The UA sensory receptors play an important role in maintaining UA patency and contribute to arousal. An impairment of UA sensory function could therefore predispose to UA obstruction during sleep. However, skeletal muscles possess a remarkable capacity of regeneration, mutability, and recovery following injury, disuse, and denervation. There are copious data, particularly in stroke field, demonstrating that physiological levels of electrical stimulation reduce muscular atrophy and dysfunction. However, owing to anatomical location and low approval by OSA patient, pharynx and genioglossus are less suitable to regular electrical stimulation. Since C-PAP is not curative, the challenge in OSA is to develop strategies to prevent and reverse the fundamental pathology and progressive UA deterioration, without side effects of drugs, and has maximal patient approval and cooperation. The current paradigm presents such a therapeutic modality.

## 3. Upper Airway Pressure and Sensory Receptors

An area of great interest and importance in nervous system mechanisms is in the generation of adequate sensory stimulation through somatosensory receptors, thus enhancing associated relevant motor functions. Several workers have successfully utilized mechanical stimulation to the soles in order to stimulate specific muscles, resulting in limb muscle contraction, thus showing that controlled sensory stimulation may be used to “drive and upregulate" muscle activation and to maintain their functional integrity. Appropriate sensory stimulation has been shown to help stem the neurophysiological processes associated with muscle unloading in no-gravity flight, and in neurogenic muscular diseases. Similarly, intraoral, tongue, and pharyngeal sensory stimulation in OSA patients may conceivably ameliorate pharyngeal and genioglossal motor dysfunction and attenuate pharyngeal collapse. 

**Figure 1:**
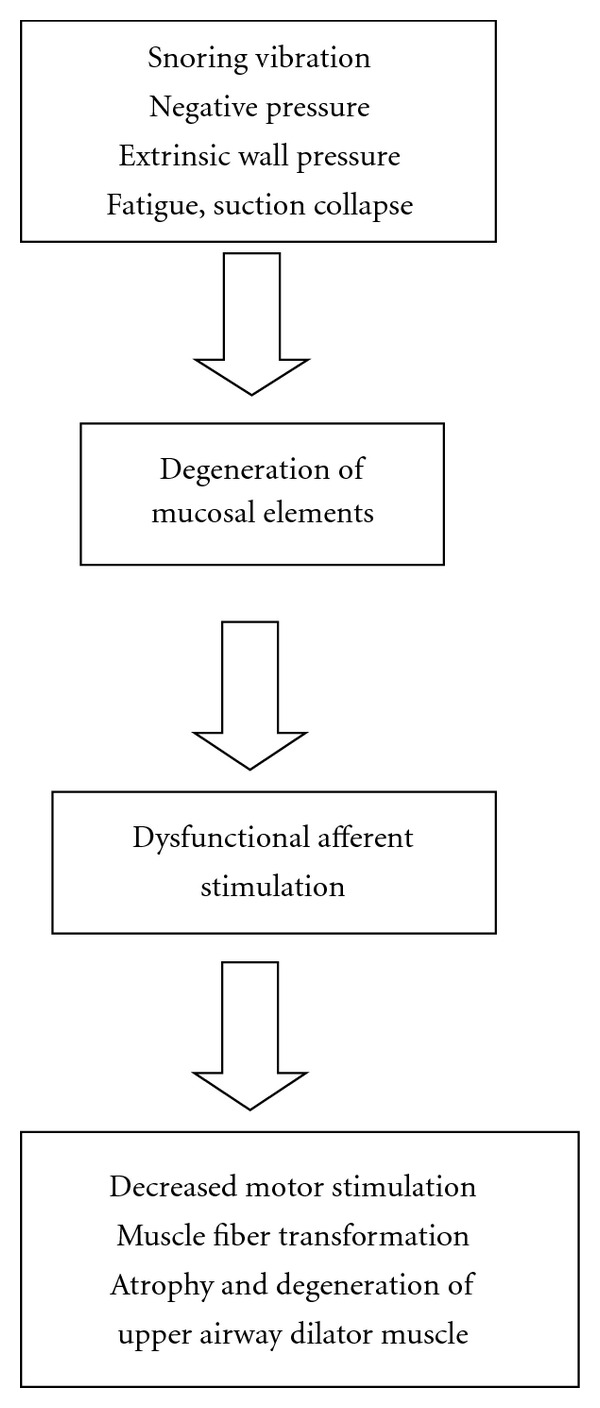
Schematic representation of dysfunctional afferent input from the periphery and consequent deleterious impact on efferent motor limb of the input-output reflex to upper airway dilator muscles, including pharynx, genioglossus, and tensor palatini.

Sensory input is critical to swallowing—a function that involves intraoral and pharyngeal muscles. Stimulation of sensory receptors participates in initiating bolus preparation of food, appropriate positioning of oral structures, modulation of the strength, velocity, and timing of muscle contractions, and finally facilitating the deglutination. In the case of UA, its muscle activity is stimulated by changes in transmural pressure; any negative transmural pressure, across the wall, therefore, is an important sensory signal, indicating that the UA is narrowed or obstructed. Thus UA negative pressure (UANP), detected by mechanoreceptors (mucosal), evokes an efferent motor reflex response that increases UA muscle activity, and stabilizes the airway [6–8]. Moreover, it is known that the discharge of many pressure-sensitive airway mechanoreceptors is influenced by the activity of UA-striated muscles [9–14]. It is understandable then that UA relaxation reduces the sensitivity of airway mechano- and other sensory receptors. It has been proposed that loss of muscle tension alters the excitability of UA motoneurons [15], and reduces tonic excitatory input from spindles of dilator muscle [16, 17], from proprioceptors [15] and/or other mechano- and sensory receptors whose activity is modulated by skeletal muscle tension. Here I have approached the problem of UA collapsibility and lack of muscular tonicity in pharyngeal constrictors, genioglossus, and TP by highlighting that the UANP is due to a reduction in sensitivity of sensory receptors in UA negative pressure. The reflex UA motor response to UANP is attenuated owing to dysfunctional sensory-motor physiological pathway. This hypothesis is shown in Figure 1.

## 4. Pathological Alterations in OSA

OSA patients snore and suffer from repeated forceful suction collapse of the pharynx and vigorous snoring-related vibration. These factors are traumatic to the UA mucosa and produce inflammation, edema, disturbed sensory function, and neurogenic damage. Habitual snoring, therefore, is considered to be a prelude to OSA. There is copious evidence of denervation of UA muscles in OSA patients. Various studies have documented demyelination [18], muscle fiber type-grouping and grouped atrophy [19, 20], clusters of N-CAM-positive fibers (marker of denervation), and axonal proliferation [21]. The palatopharyngeal muscle of OSA patients who had undergone uvulopalatopharyngoplasty also had neurogenic damage. This neural damage that is, the peripheral nerve injury results from persistent and recurring low-frequency vibration [22]. The UA muscles undergo significant inflammation, characterized by infiltration of predominantly activated CD4^+^T helper lymphocytes. The inflammatory infiltrate produces proinflammatory cytokines in response to muscle atrophy/degeneration. This triggers a cascade of inflammatory cell activation and migration into the affected muscles; the muscle undergoes further degeneration, weakness, and floppiness, due to proinflammatory cytokines, oxygen-free radicals, and nitric oxide [23–27]. 

The mucosal morphology of pharynx, uvula, and soft palate, has been characterized in OSA patients. The UA mucosa in OSA patients and snorers is described as inflammatory, edematous, and hyperplastic. Uvulas obtained from uvulopalatopharyngoplasty (UPPP) patients with moderate OSA showed a significantly higher number of leukocytes in the lamina propria of their uvular mucosa than in the controls (179 versus control 71). The thickness of the lamina propria (an index of interstitial edema) also increases significantly in OSA patients (0.99 mm versus control 0.27 mm). Hence inflammation, characterized by plasma cell infiltration and interstitial edema, present in the uvula mucosa of patients with moderate OSA, clearly suggests soft palate inflammation, contributing to UA occlusion in OSA patients [28]. Morphometry of the uvulas from UPPP OSA patients and 9 nonapneic snorers showed that both types had a significantly greater percentage of intercellular space (an index of interstitial edema) (65.7% versus control 54.0%). The covering epithelium was significantly thicker in OSA patients and snorers than in the controls. Light microscopy of both apneics and snorers revealed mucous gland hypertrophy with ductal dilation and focal squamous metaplasia, disruption of muscle bundles by infiltrating mucous glands, focal atrophy of muscle fibers, and extensive edema of the lamina propria with vascular dilatation. Severe snorers did not differ qualitatively from apneics in the characteristic changes found; however, some snorers had less extensive changes. Electron microscopy of apneics' mucosa identified frequent focal degeneration of myelinated nerve fibers and axons. Degenerative changes in peripheral nerves, however, may conceivably contribute to the development of obstructive apnea by impairing sensory-motor reflex [18]. 

Mucosal sensory dysfunction is present at multiple UA sites in patients with OSA. Utilizing air-pressure pulses endoscopic testing, a significant impairment in sensory detection thresholds in oropharynx, velopharynx, hypopharynx, and larynx (aryepiglottic eminence) has been determined. Further, *∼*61% of OSA subjects possess abnormal laryngeal sensation also, showing that mucosal sensory function is impaired at multiple UA foci in OSA [29]. Determination of temperature thresholds on the skin is an established method of detecting a neuropathy. The OSA patients showed a statistically significant higher threshold for warmth on the anterior tonsillar pillar (46.8°C versus 42.5°C control) and the tip of the tongue (40.1°C versus control 38.2°C). Consequently, patients with OSA seemingly suffer from a neuropathy in the pharynx caused by repeated trauma to the pharyngeal structures from snoring vibration and/or the pharyngeal collapse during apneas, repeatedly hundreds of time each night. A neuropathy may interfere with the normal stabilizing function of the local pharyngeal neuromuscular reflex mechanism and cause UA collapse during inspiration [30]. The two-point discrimination (2PD) and vibratory sensation threshold (VT) measurements in OSA showed the presence of a selective impairment in the detection of sensory stimuli in the UA of OSA patients as well as nonapneic snorers [22, 31]. All above-mentioned neural abnormalities are expected to exacerbate by ongoing hypoxic/hypoxemic damage during recurring apneas each night.

## 5. Mutability of Muscle Fiber Types: Loss of Aerobic Tonic Type I Muscle Fibers in Upper Airway Dilator Muscles in OSA

 The decreased sensory afferent input, a narrower pharyngeal lumen, and negative pressure may all enhance the efferent motor output to the hyomandibular muscles as a compensatory mechanism to balance the dilator genioglossal dysfunction; this would be an effort to overcome hypoventilation as well as impending pharyngeal collapse. Thus, all dilators including the anterior hyoid muscles in conjunction with the genioglossus and tensor veli palitini [32–34] may function in concert to enhance pharyngeal pressure. It is reasonable to expect that a central efferent motor output to these major and minor dilator muscles may trigger tonic activity to enhance and maintain pharyngeal patency at higher apnea hypopnea index (AHI). However, physiologically, this is not the case in OSA patients. Leiter et al. [35] posit that depending on the nature of airflow, the pharynx may not always reflexly activate the genioglossus. If this argument is true, it would mean that regardless of the level of decrease in genioglossus dilatory activity, stimulation of other neurophysiologically relevant dilator muscles, such as the hyomandibular muscles, may contribute and provide reflex compensatory motor function to dilate and stiffen the pharynx in OSA [34]. At higher AHI, these muscles are incapable of providing sustained tonic activation to the dilators; the anterior hyoid muscles may fire only phasically, providing only brief force-generation [36]. The reason for the predominantly phasic contraction of these muscles is due to transformation of their slow-twitch muscle fibers to fast-twitch anaerobic variety as described below.

 It is well established that skeletal muscle fibers are versatile and inherently mutable; they may undergo transformation in response to appropriate stimuli. They may shift from being slow-twitch oxidative Type I to fast-twitch glycolytic Type II fibers, or vice versa. While the former fiber type is more suited to sustained contractility owing to their inherent fatigue resistance, the fast-twitch Type II fibers, although generate increased force as brief bursts, are more prone to fatigue. An increase in fast-twitch type II fibers was shown in the genioglossus muscle of OSA patients [37]; this shift toward anaerobic fast-twitch glycolytic Type II fiber variety was also reported by others [38]. Carrera et al. [39, 40] studied the characteristics of the genioglossus in OSA patients (versus controls) and reported a Type II fast-twitch fiber predominance and enhanced muscle fatigability. Indeed, changes similar to those in humans also occur in animal models. A shift toward fast-twitch glycolytic Type II fiber in the sternohyoid and geniohyoid muscles in the English bulldog, used as an animal model of OSA, has been documented [41, 42]. In the rat, both short- and longer-term intermittent hypoxia have been shown to produce glycolytic shifts as fast-twitch fast-fatigue type II fibers, and hence the increased fatigability in their geniohyoid and sternohyoid muscles [43, 44]. Thus, intermittent hypoxia in OSA has the propensity to transform muscle fibers to fast-twitch and fast-fatigue fiber type variety in the dilator muscles, including those of the hyomandibular muscle complex in the OSA patients, as emphasized recently [36]. Given the degree and type of phasic contractile function plus the fatigueability, and the presence of inflammation, neurogenic atrophy, and degeneration in muscle fibers of the dilators, they are unable to produce tonic sustained force generation to overcome repeated apnea, UA collapse, and dysfunctional breathing.

## 6. Discussion

 Aging-related disabilities and diseases are caused by increasing human longevity. Aging causes a general decrease in a variety of functional loops subserving somatosensory, special sensory, perceptual and cognitive operations. Losses in various sensory modalities include vision, auditory, gustatory, olfactory, vestibular, and indeed somatosensory. Over 35% of elderly have hearing deficits. About 11% elderly have noncorrectable visual impairment. In excess of 50% of elderly possess olfactory impairment. The consequence of intrinsic aging and extrinsic environmental insult results in progressive atrophy of dermal and epidermal sensory structures of the tongue, thus compromising the gustatory sense; this has been abundantly documented in geriatric population. Similarly, UA sensory decline occurring in the UA mucosal sensory receptors, as pointed out above, in over 10% of healthy elderly population, comprises part of a global sensory decline in aging [1, 45, 46]. 

### 6.1. Upper Airway (UA) and OSA

Normal breathing is closely linked with patency of UA. The UA dilator genioglossus muscle plays an essential role in maintaining UA patency. Incidence of sleep-disordered breathing increasing with age and correlated with UA collapsibility, has been documented in old animals also [41, 42]. In OSA, vigorous snoring-related vibration and repeated forceful suction collapse of the pharynx at night are traumatic to the UA mucosa and has been shown to produce inflammation, edema, sensory perturbations, and neurogenic damage [19, 21–26, 28, 31, 47]. A significant impairment in sensory threshold occurs in OSA (versus control subjects) in all pharyngeal components including oropharynx, velopharynx, and hypopharynx. With aging, in addition to sensory decline [46], there are changes in motor nerve axon, including axonal flow, axon terminals, end plate morpho-physiology, and synaptic dysfunction, leading to overall neuromuscular pathology [18, 20, 27, 29, 48–51]. The above-mentioned pathological perturbations are universal in cranial and noncranial sensorimotor system during senescence. The documented neural-muscular abnormalities are expected to be further exacerbated by ongoing potential hypoxic/hypoxemic damage from repeated apnea [1, 52]. 

 OSA is a specific entity and the apnea-hypopnea index (AHI) defines the degree of severity of this disorder. About 10% of elderly population has an AHI of ≥10. 152 “healthy older adult volunteers without any disease”, (Mean age of 66) except for “Undiagnosed sleep-related breathing disorders (SRBD)” plus an absence of subjective daytime sleepiness, and an AHI of 21 (±15) for males, and 16.7 (±12.9) for females, respectively, were studied in a french study [53]. Whole brain gray matter volume (GMV) scans did not differ significantly between subjects with and without SRBD; however, bilaterally, a significant inverse relationship was found between AHI and GMV in some nuclei of the pontomedullary reticular zone—these included the dorsal nucleus of the vagus, the solitary tract nucleus, and the nucleus ambiguus [53]. Thus symmetrical atrophy of these key brainstem nuclei is a function of possible compromises in several sensory modalities including the somatosensory and gustatory. The above-mentioned brainstem nuclei of the ponto-medullary junction, undergoing atrophy, conceivably impact on hypoglossal nucleus, which in turn causes genioglossus dysfunction. The activity decline in this important dilator muscle has been shown to interfere with UA patency, causing recurrent hypoxia, hypoxemia, and hypercapnia. These physiological alterations have a wide ranging repercussion including interference with cerebrovascular function, regional cerebral blood flow, and cognitive functions (see [1]). The link between sensory decline and dysfunctional sleep with cardiorespiratory and cognitive dysfunction demands that we focus on developing novel interventional and therapeutic strategies to prevent commencement and progression of this indolent and serious human disorder.

Advances in brain imaging have given many insights into the cortical and subcortical control of human UA and pharyngeal function. UA patency represents a complex coordinated process that is dependent on sensory feedback [54–60]. The UA collapse is seen as a neurologic disorder owing to impaired peripheral sensory afferents affecting UA muscular structures. There are copious data supporting the above contention—ranging from effects of functional oropharyngeal disruption on the cortical control of pharynx in swallowing [56–58] to oropharyngeal anesthesia that causes reduced sensory input which leads to aspiration and impaired motor efferents to the pharynx [61, 62]. Further, it highlights that a short-term decrease in oropharyngeal sensory input leading to diminished cortical sensory activity is associated with reduced activation of the primary motor cortex. This underlines the important role of sensory information on the cortical coordination of human pharyngeal motor functions involved in both swallowing and UA patency [63]. Average fMRI blood oxygenation level-dependent (BOLD) signal intensity and number of activated voxels during swallowing concurrent with gustatory, and olfactory stimulations were significantly increased throughout the cortical swallowing/pharyngeal network. In addition to other relevant regions, there was increased activity in sensory-motor cortex [64]. It is anticipated, therefore, that simple nutritive gustatory-sensory stimulation may also have clinical implications in the management of pharyngeal collapse in OSA.

Several studies have examined the effect of oropharyngeal stimulation utilizing either thermal or taste stimuli to trigger a more rapid response of pharynx. Cold stimulation of the anterior faucial pillars (AFP) before swallowing hastened the onset of the pharyngeal swallowing phase and reduced the swallowing latency [57, 65, 66]. Tactile-thermal-oral stimulation on patients with neurologic diseases results in improved triggering of the pharyngeal function (see [54, 57]). The AFP is a component of the soft palate and is found bilaterally on the oral side of the velum. They are innervated by the maxillary branch of the trigeminal nerve and the glossopharyngeal nerve and can be easily stimulated by thermal and tactile activation [54]. Also taste stimuli have been shown to enhance pharyngeal function indicating that afferents from disparate oral-pharyngeal sensory receptors can facilitate pharyngeal muscular upregulation. The following section delineates the above mentioned further.

### 6.2. Changes in Upper Airway Dilator Muscle Function in OSA

OSA is a state-related disorder, and the most fundamental neural event that initiates OSA is the closure of the pharyngeal airway during sleep. UA dilator muscle activity maintains a patent airway during wakefulness; however, with sleep onset, this neural compensation is reduced [67], thus setting the stage for OSA. During sleep, cortical neural control—that includes changes in neural drive, and peripheral reflex control of the chest wall muscles, UA dilator muscles, and ventilation, is compromised [67, 68]. Several conditions such as obesity, increased neck size, and a host of other risk factors (including changes in craniofacial structures particularly retrognathia and enlargement of the UA soft tissue structures) would alter UA anatomy. However, any abnormal UA anatomy impinging on retropalatal and/or retroglossal regions would interfere with UA dilator and pharyngeal constrictor muscles, thus resulting in a nocturnal reduction in motor activity of these muscles [69–71]. Individuals afflicted with sleep apnea have a more narrow and collapsible pharynx than normal persons, and that UA obstruction *per se* is an essential prerequisite for the development of OSA [72, 73]. Neural control/UA factors occupy a central position in either enhancing or attenuating the OSA—in keeping with the functional status of sensory-motor reflexes, emphasized here. Therefore, the major therapeutic challenge is to keep these reflexes and UA functional when the OSA patient is asleep. In this regard, the current paradigm may possess the potential to accomplish this and open new vistas in possible novel therapeutic strategies. 

The UA is most vulnerable behind the tongue base and the soft palate that lack skeletal support. It is kept patent by a number of factors notably the motor activation of the dilator muscles. Sleep onset is characterized by ventilation decreases, and several studies have confirmed that the UA of OSA patients is more collapsible than the UA of nonapneic control subjects [74]. At sleep onset, control subjects showed small but consistent decrements in the activity of both the TP and GG muscles [75]; however, OSA patients demonstrated significantly greater decrements in TP activity, evidenced by EMG [76–78]. The effect of sleep onset on GG EMG in OSA patients was inconsistent and heterogeneous, although most patients showed decrements in GG activity, significantly larger than controls [79]. The decrements in TP and GG muscles' activity in sleep in OSA patient reflect a loss of neuromuscular function that exists during wakefulness [79]. Further, obstructive respiratory events are more common and prolonged during REM sleep; therefore, documented decreases in activity of TP and GG during REM sleep correlate with increases in the UA resistance that precipitates UA collapse and OSA [80, 81]. 

UA patency is a function of neural control of dilator muscles; UA collapse involves not only increased intrapharyngeal negative pressure, but dysfunctional receptor types, including vagal pulmonary receptors, UA mechanoreceptors, other somatosensory receptors, and olfactory and gustatory receptors. Edema and inflammation of pharyngeal tissues, due to snoring plus suction collapse, might cause dysfunction of the above-mentioned receptors (normally responsible for protective reflexes) and cause narrowing of the UA. UA collapsibility in OSA patients has been shown to correlate with changes in tonic and phasic contraction of UA dilator muscles, that is, TP and GG, respectively. Decreases in GG (EMG) activation occurs during REM in normal subjects [82], in OSA patients [83], and in sleeping dogs [84]. At sleep onset and maintained sleep state, TP continues to have low activity [75] and responds mainly to rapid pulses of negative pressure [85]; furthermore, the TP baseline activity was markedly reduced during Non-REM sleep as compared with wakefulness [85]. Hence UA neuromuscular facilitation during sleep is defective in OSA events. Unlike GG, TP is a tonic UA muscle [86]. In normal controls [75] and OSA patients [87], there is diminution in tonic activation of both GG and TP muscles at sleep onset; however, this depletion is particularly more pronounced in the predominantly tonic TP [75, 88, 89]. Interestingly, during respiratory events in REM sleep, the tonic activity of GG reduces more than that noted in NREM sleep (stage 2 sleep) [81]. Consistently shown has been a fall in TP activity in stable sleep than in wakefulness [75–78, 87–89]. In conjunction with GG dysfunction, decreased tonic TP activity is considered contributory to predisposition to airway collapse [81]. It is, therefore, logical that appropriate measures are applied in OSA patients, to increase not only phasic GG activity, but equally important-tonic TP function as well.

### 6.3. Stimulation of Disparate Sensory Modalities

Sensory input is crucial to the initiation and modulation of several pharyngeal functions, and integration of diverse cortical and brainstem networks that underpin sensory-motor function in the UA. The oral cavity and pharynx are functionally integrated regions involved in complex motor responses that include feeding, chewing, swallowing, speech, and respiration. A variety of sensory receptors that innervate these two regions provide the link in reflexes that control muscles of the UA and upper gastrointestinal tract. The quintessential reflexes affect the neural motor control in general [90, 91] and diverse tongue muscles by motoneurons in the hypoglossal nucleus, in particular [92].

Several interesting studies have determined effects of functional oropharyngeal sensory disruption on the pharyngeal motor function (swallowing), that is, with and without topical oropharyngeal anesthesia (in healthy subjects). Unlike the normal bilateral activation of the primary sensorimotor cortex, oropharyngeal anesthesia led to a pronounced decrease of both sensory and motor activation. This showed that a short-term decrease in oropharyngeal sensory input impedes the control of pharyngeal function [56, 59]. Thermal tactile oral stimulation (TTOS) is a method used to treat patients with neurogenic dysphagia caused by sensory deficits. Recently Teismann et al. [57, 60] studied pharyngeal function in healthy subjects with and without TTOS. Synthetic aperture magnetometric (SAM) analysis of data showed that TTOS facilitated both the oral and the pharyngeal phases of deglutition. The pharyngeal function has been shown to improve following mechanical + cold + gustatory stimulation to the human anterior faucial pillars (AFP) [93]. Similarly, light touch to the AFP with a warm (body temperature) or cold probe also evoked a significant pharyngeal response [54, 66]. Many studies in the literature show that sensory receptors of soft palate, pharyngeal surface of epiglottis, glossoepiglottic sinus, posterior pharyngeal wall, and the pharyngo-esophageal junctions may be utilized as potential sites for pharyngeal stimulation. Excitation of the mucosal water receptors (with distilled water) of the pharyngeal region and excitation of the gustatory receptors for salt (with 0.3 M NaCl solution) in the posterior tongue showed that both types of sensory inputs enhanced the pharyngeal function, measured by submental electromyography [94]. Similarly, stimulation of the pharyngolaryngeal region with sour solutions (acetic acid or citric acid) facilitated pharyngeal function via gustatory reflex, suggesting that the enhancement in pharyngeal function may be due to increases of sensory input via the superior laryngeal nerve and the glossopharyngeal nerve [95]. A rehabilitation paradigm of thermal stimulation employed in functional recovery of limbs after acute stroke [96–98] has been utilized in order to enhance pharyngeal function also in stroke patients [98]. In dysphagic stroke subjects, tactile plus thermal application reduced duration of pharyngeal stage transition and total swallow duration [98–100]. Studies have been reported in rhesus monkey that directly evaluated the effect of sensory stimulation in pharynx. The palatopharyngeus and pharyngeal constrictor muscles were studied by direct observation with a flexible fiberoptic scope and by electromyography [101]. Placement of water in the hypopharynx produced a tonic discharge in muscle fibers of the middle and superior pharyngeal constrictors; this motor response converted to rhythmic discharge by simply obstructing the nasal cavity [101]. Further, other work in animals is noteworthy in that concurrent stimulation from different modalities potentiates summation, and the concurrent inputs enhance the pharyngeal output [102]. The imaging data highlight the importance of sensory stimulation, by showing that oro-pharyngeal air pulse stimulation augments sensory input, engages both brainstem and cerebral control centers, and activates both sensory and motor areas [103–105]. This point is particularly germane to the current hypothesis. Several other studies have shown that pharyngeal stimulation increases motor excitability, and even short-term sensory stimulation of the pharynx can induce sustained upregulation in corticobulbar pathways [106–108]. The above studies support the current hypothesis that stimulation of various UA sensory receptors may enhance central feedback resulting in increased inspiratory drive/effort, increased activity of UA dilator muscles, and decreased pharyngeal and closing pressure in OSA patients. 

The primary olfactory pathway is one of the most unique and plastic in the nervous system with neurogenesis and constant turnover of new sensory neurons. The olfactory receptors are known to turnover approximately every 40 days. New sensory neurons in the olfactory epithelium and new interneurons in the olfactory bulb (OB) are added throughout adult life of humans (and animals). This results in continuous remodeling of neuronal circuits and synapses in the OB throughout life. In humans, the trigeminal and olfactory nerves share overlapping innervation areas in the nasal cavity and function interactively. Odors perceived through the nose are called “orthonasally” perceived, whereas odors perceived through the mouth are labelled “retronasally” perceived. The latter can be used in older individuals, anosmics, and those with chronic nasal obstruction. Both routes of delivery produce similar brain activation of several brain areas, notably the amygdala and the insula; both reciprocally project to NTS and the dorsal motor nucleus [109–112]. It is well documented that the loss of olfactory function leads to decreased trigeminal sensitivity. Thus effective olfactory stimulation is a powerful tool that may be valuable in enhancing trigeminal excitation. Olfactory dysfunction is a common feature in obese [113–115], older persons [116], and old mice [117]; they possess a higher thresholds for smell. Many OSA patients happen to be older and obese. Upregulation of olfactory function has many anabolic effects on whole-body physiological functions including metabolic control and homeostasis [118] and enhanced synaptic modulation in several brain regions [119].

The pharyngeal UA afferent inputs, mediated by the trigeminal (V), facial (V11), glossopharyngeal (IX), and vagus (X) cranial nerves, terminate in the nucleus of the Solitary Tract (NTS) and the trigeminal nucleus [120]. The NTS is an obligatory relay for gustatory and several other sensory afferent inputs [121, 122]. It is the first CNS site for synaptic contact of the primary afferent fibers from the lungs as well. The afferent nerve fibers via above-mentioned cranial nerves project from the pharynx and tongue to the NTS, and synapse with second order sensory neurons in this nucleus. Thus the NTS neurons receive inputs from several receptor types from tongue and other oro-pharyngo-laryngeal regions [121, 122]. Fibers from the NTS ascend to the thalamus and then project to the insula (gustatory cortex) and the amygdala, which in turn have reciprocal projections to the NTS; thus responses to gustatory stimulation can be recorded from both NTS and the insular cortex [123]. Furthermore, there are direct projections from the principal trigeminal sensory nucleus [124–127], and from NTS [128], to the hypoglossal nucleus. Thus the signal processing at the NTS determines several reflex outcomes. 

OSA is characterized by sensory receptor dysfunction, which adversely impacts several brainstem nuclei including trigeminal, NTS, and hypoglossal. Conceivably, all receptors may not become dysfunctional in all components of the UA, and the OSA patients do possess functional sensory-motor reflex and afferent discharges found in arousal and wakefulness. Consequently, a combination of diurnal sensory stimulation protocols encompassing olfactory, gustatory, and different types of somatosensory (e.g., tactile, temperature, and pressure) need to abe applied, in a unisensory (unimodal) and multisensory (multimodal) manner; conceivably this should strengthen and upregulate the diverse sensory receptors, enhance peripheral and central neuronal excitability/synaptic transmission, causing an increase in genioglossal function. Several studies described above show that the multisensory stimulation is more effective than just the unimodal.

### 6.4. Role of Neural Plasticity

There is a remarkable degree of plasticity in primary motor cortex (M1) and the somatosensory cortex, as abundantly documented by the whisker barrel cortex studies. Some plastic changes occur very early; for example, M1 neurons in primates can be activated by a tactile and proprioceptive input with only a short latency [129]. Interestingly, even under anesthesia, the trigeminal afferents participate in activating M1 neurons, via primary sensory cortex → to M1 associational connections. There is a large literature on the effect of somatosensory stimulation, utilizing electrical, tactile, and thermal modalities, on improving motor functions in stroke patients (several have been referred to above), and readers are suggested to look up these data on clinical rehabilitation through sensory application. Other options available include repetitive transcranial magnetic stimulation of the motor cortex pinpointing the UA motor region in OSA, as is done in spasticity. Alternatively, functional motor recovery in the UA dilator muscles may be accomplished noninvasively by bedside simple easy-to-do thermal, proprioceptive, and air puff-related somatosensory and gustatory stimulation [130, 131]. These points are delineated in the above sections. Finally, genioglossus activity is not different between OSA patients on CPAP and healthy controls during stable NREM sleep [132]. Therefore, the mechanism driving the increased dilator muscle activity during wakefulness in OSA patients is lost in sleep. This particular mechanism conceivably involves some type of neural plasticity which is sensitive to sleep, causing the genesis of UA negative pressure. Therefore, an enhancement of excitatory trains of sensory stimuli in wakefulness/sleep may attenuate impairment of dilator muscle function in sleep; this may have a good therapeutic impact and provide an important UA rehabilitation outcome in OSA. Finally, it is only logical to endeavor and stimulate different easily amenable peripheral receptors in the UA in a novel noninvasive nonpharmacological manner to ameliorate OSA. We need to first establish the safest and most efficacious combination of the available sensory modalities as the treatment strategy. The insight delineated here is likely to be profoundly important for treatment of OSA. 

## 7. Conclusions

Despite an extraordinary degree of research in understanding OSA, we still lack a satisfactory insight into the early stages of neuropathogenesis of this common condition. The quest for testable hypotheses is ongoing, and efforts to ameliorate pathogenetic stigmata persist. The present paper seeks to highlight the importance of damage in peripheral sensory elements that cause dysfunction in the afferent portion of the “sensory → motor” loop; the resulting dysfunctional motor efferent responses, may be an equally critical factor in the genesis of recurrent UA collapse—causing repeated hypoxic, hypoxemic, and hypercapnic events in OSA. The logical and pragmatic approach, therefore, is to upregulate the dysfunctional sensory receptors, via sensory stimulation, preferably in a natural, physiological, nonpharmacologic, and noninvasive manner from the periphery (i.e., intraoral, genioglossal, and proximal pharyngeal regions), in order to stimulate motor output limb and ameliorate the pathology of dilator muscles, including genioglossus and TP. Such ongoing upregulation of sensory → motor loop should regenerate the dysfunctional receptors and enhance the activity of those mucosal receptors that are intact; this conceivably should strengthen UA muscles, decrease mechanical load, and produce nocturnal UA patency through contractility of the dilators' aerobic tonic type 1, tonic-phasic mainly oxidative type 2, and mainly phasic glycolytic type 2 muscle fibers. Further research is critically important focusing on harnessing the role of disparate multimodal afferent signals encompassing somatosensory, olfactory, and gustatory modalities in upregulating key brainstem nuclei, and mediating activation of the motor pathway in possible amelioration of OSA.

## References

[1] Daulatzai MA (2010). Early stages of pathogenesis in memory impairment during normal senescence and alzheimer's disease. *Journal of Alzheimer's Disease*.

[2] Daulatzai MA (2010). Conversion of elderly to Alzheimer's dementia: role of confluence of hypothermia and senescent stigmata—the plausible pathway. *Journal of Alzheimer's Disease*.

[3] Blitz DM, Beenhakker MP, Nusbaum MP (2004). Different sensory systems share projection neurons but elicit distinct motor patterns. *Journal of Neuroscience*.

[4] Büschges A, El Manira A (1998). Sensory pathways and their modulation in the control of locomotion. *Current Opinion in Neurobiology*.

[5] Daulatzai MA, Khandoker A (2008). Therapeutic strategy in attenuating obstructive sleep apnea by sensory neurological stimulation modalities. *Sleep and Biological Rhythms*.

[6] Amis TC, O’Neill N, Wheatley JR, van der Touw T, Di Somma E, Brancatisano A (1999). Soft palate muscle responses to negative upper airway pressure. *Journal of Applied Physiology*.

[7] Mathew OP, Sant’Ambrogio G, Fisher JT, Sant’Ambrogio FB (1984). Laryngeal pressure receptors. *Respiration Physiology*.

[8] Mathew OP, Santambrogio G, Fisher JT, Santambrogio FB (1984). Respiratory afferent activity in the superior laryngeal nerves. *Respiration Physiology*.

[9] Sant’Ambrogio G, Mathew OP, Fisher JT, Sant’Ambrogio FB (1983). Laryngeal receptors responding to transmural pressure, airflow and local muscle activity. *Respiration Physiology*.

[10] Sant’Ambrogio G, Mathew OP, Sant’Ambrogio FB (1985). Role of intrinsic muscles and tracheal motion in modulating laryngeal receptors. *Respiration Physiology*.

[11] Sant’Ambrogio G, Tsubone H, Sant’Ambrogio FB (1995). Sensory information from the upper airway: role in the control of breathing. *Respiration Physiology*.

[12] Sekizawa S, Tsubone H (1991). The respiratory activity of the superior laryngeal nerve in the rat. *Respiration Physiology*.

[13] Sekizawa SI, Tsubone H, Hishida N, Kuwahara M, Sugano S (1997). The Afferent Activity of the Superior Laryngeal Nerve, and Respiratory Reflexes Specifically Responding to Intralaryngeal Pressure Changes in Anesthetized Shiba Goats. *Journal of Veterinary Medical Science*.

[14] Tsubone H, Mathew OP, Sant’Ambrogio G (1987). Respiratory activity in the superior laryngeal nerve of the rabbit. *Respiration Physiology*.

[15] Janczewski WA (1997). Muscle relaxation attenuates the reflex response to laryngeal negative pressure. *Respiration Physiology*.

[16] Rozhold O (1980). Muscle spindles in the external muscles of the human tongue. *Acta Universitatis Palackianae Olomucensis Facultatis Medicae*.

[17] Smith KK (1989). Histological demonstration of muscle spindles in the tongue of the rat. *Archives of Oral Biology*.

[18] Woodson BT, Garancis JC, Toohill RJ (1991). Histopathologic changes in snoring and obstructive sleep apnea syndrome. *Laryngoscope*.

[19] Edstrom L, Larsson H, Larsson L (1992). Neurogenic effects on the palatopharyngeal muscle in patients with obstructive sleep apnoea: a muscle biopsy study. *Journal of Neurology Neurosurgery and Psychiatry*.

[20] Lindman R, Stål PS (2002). Abnormal palatopharyngeal muscle morphology in sleep-disordered breathing. *Journal of the Neurological Sciences*.

[21] Boyd JH, Petrof BJ, Hamid Q, Fraser R, Kimoff RJ (2004). Upper airway muscle inflammation and denervation changes in obstructive sleep apnea. *American Journal of Respiratory and Critical Care Medicine*.

[22] Kimoff RJ, Sforza E, Champagne V, Ofiara L, Gendron D (2001). Upper airway sensation in snoring and obstructive sleep apnea. *American Journal of Respiratory and Critical Care Medicine*.

[23] Créange A, Lefaucheur JP, Authier FJ, Gherardi RK (1998). Cytokines and peripheral neuropathiesCytokines et neuropathies peripheriques. *Revue Neurologique*.

[24] Harvey GK, Gold R, Hartung HP, Toyka KV (1995). Non-neural-specific T lymphocytes can orchestrate inflammatory peripheral neuropathy. *Brain*.

[25] Reid MB, Lännergren J, Westerblad H (2002). Respiratory and limb muscle weakness induced by tumor necrosis factor-*α*: involvement of muscle myofilaments. *American Journal of Respiratory and Critical Care Medicine*.

[26] Shindoh C, Hida W, Ohkawara Y (1995). TNF-*α* mRNA expression in diaphragm muscle after endotoxin administration. *American Journal of Respiratory and Critical Care Medicine*.

[27] Spies JM, Westland KW, Bonner JG, Pollard JD (1995). Intraneural activated T cells cause focal breakdown of the blood-nerve barrier. *Brain*.

[28] Sekosan M, Zakkar M, Wenig BL, Olopade CO, Rubinstein I (1996). Inflammation in the uvula mucosa of patients with obstructive sleep apnea. *Laryngoscope*.

[29] Nguyen ATD, Jobin V, Payne R, Beauregard J, Naor N, Kimoff RJ (2005). Laryngeal and velopharyngeal sensory impairment in obstructive sleep apnea. *Sleep*.

[30] Larsson H, Carlsson-Nordlander B, Lindblad LE, Norbeck O, Svanborg E (1992). Temperature thresholds in the oropharynx of patients with obstructive sleep apnea syndrome. *American Review of Respiratory Disease*.

[31] Jobin V, Champagne V, Beauregard J, Charbonneau I, McFarland DH, Kimoff RJ (2007). Swallowing function and upper airway sensation in obstructive sleep apnea. *Journal of Applied Physiology*.

[32] Eckert DJ, Malhotra A (2008). Pathophysiology of adult obstructive sleep apnea. *Proceedings of the American Thoracic Society*.

[33] Horner RL, Innes JA, Morrell MJ, Shea SA, Guz A (1994). The effect of sleep on reflex genioglossus muscle activation by stimuli of negative airway pressure in humans. *Journal of Physiology*.

[34] Palmer PM, Luschei ES, Jaffe D, McCulloch TM (1999). Contributions of individual muscles to the submental surface electromyogram during swallowing. *Journal of Speech, Language, and Hearing Research*.

[35] Leiter JC, Knuth SL, Bartlett D (1992). Dependence of pharyngeal resistance on genioglossal EMG activity, nasal resistance, and airflow. *Journal of Applied Physiology*.

[36] Daulatzai M, Karmakar C, Khan N, Khandoker A, Palaniswami M Unravelling unique qualitative and quantitative characteristics of the surface submentalis EMG in OSA polysomnograms.

[37] Sériès F, Simoneau JA, Pierre SST, Marc I (1996). Characteristics of the genioglossus and musculus uvulae in sleep apnea hypopnea syndrome and in snorers. *American Journal of Respiratory and Critical Care Medicine*.

[38] Smirne S, Iannaccone S, Ferini-Strambi L, Comola M, Colombo E, Nemni R (1991). Muscle fibre type and habitual snoring. *Lancet*.

[39] Carrera M, Barbé F, Sauleda J, Tomás M, Gómez C, Agustí AGN (1999). Patients with obstructive sleep apnea exhibit genioglossus dysfunction that is normalized after treatment with continuous positive airway pressure. *American Journal of Respiratory and Critical Care Medicine*.

[40] Carrera M, Barbé F, Sauleda J (2004). Effects of obesity upon genioglossus structure and function in obstructive sleep apnoea. *European Respiratory Journal*.

[41] Petrof BJ, Hendricks JC, Pack AI (1996). Does upper airway muscle injury trigger a vicious cycle in obstructive sleep apnea? A hypothesis. *Sleep*.

[42] Petrof BJ, Pack AI, Kelly AM, Eby J, Hendricks JC (1994). Pharyngeal myopathy of loaded upper airway in dogs with sleep apnea. *Journal of Applied Physiology*.

[43] Bradford A, McGuire M, O’Halloran KD (2005). Does episodic hypoxia affect upper airway dilator muscle function? Implications for the pathophysiology of obstructive sleep apnoea. *Respiratory Physiology and Neurobiology*.

[44] Pae EK, Wu J, Nguyen D, Monti R, Harper RM (2005). Geniohyoid muscle properties and myosin heavy chain composition are altered after short-term intermittent hypoxic exposure. *Journal of Applied Physiology*.

[45] Appollonio I, Carabellese C, Magni E, Frattola L, Trabucchi M (1995). Sensory impairments and mortality in an elderly community population: a six-year follow-up study. *Age and Ageing*.

[46] Shaffer SW, Harrison AL (2007). Aging of the somatosensory system: a translational perspective. *Physical Therapy*.

[47] Friberg D, Gazelius B, Lindblad LE, Nordlander B (1998). Habitual snorers and sleep apnoics have abnormal vascular reactions of the soft palatal mucosa on afferent nerve stimulation. *Laryngoscope*.

[48] Connor NP, Suzuki T, Lee K, Sewall GK, Heisey DM (2002). Neuromuscular junction changes in aged rat thyroarytenoid muscle. *Annals of Otology, Rhinology and Laryngology*.

[49] Kerezoudi E, Thomas PK (1999). Influence of age on regeneration in the peripheral nervous system. *Gerontology*.

[50] Son YJ, Thompson WJ (1995). Nerve sprouting in muscle is induced and guided by processes extended by Schwann cells. *Neuron*.

[51] Son YJ, Trachtenberg JT, Thompson WJ (1996). Schwann cells induce and guide sprouting and reinnervation of neuromuscular junctions. *Trends in Neurosciences*.

[52] Daulatzai MA (2009). The pharyngeal landscape: its length and breadth. *Journal of Sleep Research*.

[53] Celle S, Peyron R, Faillenot I (2009). Undiagnosed sleep-related breathing disorders are associated with focal brainstem atrophy in the elderly. *Human Brain Mapping*.

[54] Kaatzke-McDonald MN, Post E, Davis PJ (1996). The effects of cold, touch, and chemical stimulation of the anterior faucial pillar on human swallowing. *Dysphagia*.

[55] Kimoff RJ, Cheong TH, Olha AE (1994). Mechanisms of apnea termination in obstructive sleep apnea: role of chemoreceptor and mechanoreceptor stimuli. *American Journal of Respiratory and Critical Care Medicine*.

[56] Teismann IK, Steinstraeter O, Stoeckigt K (2007). Functional oropharyngeal sensory disruption interferes with the cortical control of swallowing. *BMC Neuroscience*.

[57] Teismann IK, Steinsträter O, Warnecke T (2009). Tactile thermal oral stimulation increases the cortical representation of swallowing. *BMC Neuroscience*.

[58] Teismann IK, Dziewas R, Steinstraeter O, Pantev C (2009). Time-dependent hemispheric shift of the cortical control of volitional swallowing. *Human Brain Mapping*.

[59] Ertekin C, Kiylioglu N, Tarlaci S, Keskin A, Aydogdu I (2000). Effect of mucosal anaesthesia on oropharyngeal swallowing. *Neurogastroenterology and Motility*.

[60] Miura Y, Morita Y, Koizumi H, Shingai T (2009). Effects of taste solutions, carbonation, and cold stimulus on the power frequency content of swallowing submental surface electromyography. *Chemical Senses*.

[61] Jafari S, Prince RA, Kim DY, Paydarfar D (2003). Sensory regulation of swallowing and airway protection: a role for the internal superior laryngeal nerve in humans. *Journal of Physiology*.

[62] Sulica L, Hembree A, Blitzer A (2002). Swallowing and sensation: evaluation of deglutition in the anesthetized larynx. *Annals of Otology, Rhinology and Laryngology*.

[63] Michou E, Hamdy S (2009). Cortical input in control of swallowing. *Current Opinion in Otolaryngology and Head and Neck Surgery*.

[64] Babaei A, Kern M, Antonik S (2010). Enhancing effects of flavored nutritive stimuli on cortical swallowing network activity. *American Journal of Physiology*.

[65] Dziewas R, Sörös P, Ishii R (2005). Cortical processing of esophageal sensation is related to the representation of swallowing. *NeuroReport*.

[66] Li YQ, Takada M, Mizuno N (1993). Identification of premotor interneurons which project bilaterally to the trigeminal motor, facial or hypoglossal nuclei: a fluorescent retrograde double-labeling study in the rat. *Brain Research*.

[67] Haxhiu MA, Mack SO, Wilson CG, Feng P, Strohl KP (2003). Sleep networks and the anatomic and physiologic connections with respiratory control. *Frontiers in Bioscience*.

[68] Khoo MCK (2000). Determinants of ventilatory instability and variability. *Respiration Physiology*.

[69] Schwab RJ, Gupta KB, Gefter WB, Metzger LJ, Hoffman EA, Pack AI (1995). Upper airway soft tissue anatomy in normals and patients with sleep disordered breathing: significance of the lateral pharyngeal walls. *American Journal of Respiratory and Critical Care Medicine*.

[70] Trudo FJ, Gefter WB, Welch KC, Gupta KB, Maislin G, Schwab RJ (1998). State related changes in upper airway caliber and surrounding soft tissue structures in normals. *American Journal of Respiratory and Critical Care Medicine*.

[71] Rowley JA, Sanders CS, Zahn BR, Badr MS (2001). Effect of REM sleep on retroglossal cross-sectional area and compliance in normal subjects. *Journal of Applied Physiology*.

[72] Alex CG, Onal E, Lopata M (1986). Upper airway occlusion during sleep in patients with Cheyne-Stokes respiration. *American Review of Respiratory Disease*.

[73] Tkacova R, Niroumand M, Lorenzi-Filho G, Bradley TD (2001). Overnight shift from obstructive to central apneas in patients with heart failure: role of PCO and circulatory delay. *Circulation*.

[74] Jordan AS, White DP (2008). Pharyngeal motor control and the pathogenesis of obstructive sleep apnea. *Respiratory Physiology and Neurobiology*.

[75] Worsnop C, Kay A, Pierce R, Kim Y, Trinder J (1998). Activity of respiratory pump and upper airway muscles during sleep onset. *Journal of Applied Physiology*.

[76] Worsnop C, Kay A, Kim Y, Trinder J, Pierce R (2000). Effect of age on sleep onset-related changes in respiratory pump and upper airway muscle function. *Journal of Applied Physiology*.

[77] Fogel RB, Trinder J, Malhotra A (2003). Within-breath control of genioglossal muscle activation in humans: effect of sleep-wake state. *Journal of Physiology*.

[78] Fogel RB, Malhotra A, White DP (2004). Sleep *·* 2: pathophysiology of obstructive sleep apnoea/hypopnoea syndrome. *Thorax*.

[79] Mezzanotte WS, Tangel DJ, White DP (1996). Influence of sleep onset on upper-airway muscle activity in apnea patients versus normal controls. *American Journal of Respiratory and Critical Care Medicine*.

[80] Gaultier C (1994). Upper airway muscles and pathophysiology of obstructive sleep apnea syndromeMOTRICITE DES VOIES AERIENNES SUPERIEURES ET PHYSIOPATHOLOGIE DU SYNDROME D’APNEES DU SOMMEIL. *Neurophysiologie Clinique*.

[81] Jordan AS, Whit DP, Lo Y-L (2009). Airway dilator muscle activity and lung volume during stable breathing in obstructive sleep apnea. *Sleep*.

[82] Kuna ST, Smickley J (1988). Response of genioglossus muscle activity to nasal airway occlusion in normal sleeping adults. *Journal of Applied Physiology*.

[83] Okabe S, Hida W, Kikuchi Y, Taguchi O, Takishima T, Shirato K (1994). Upper airway muscle activity during REM and non-REM sleep of patients with obstructive apnea. *Chest*.

[84] Harms CA, Zeng YJ, Smith CA, Vidruk EH, Dempsey JA (1996). Negative pressure-induced deformation of the upper airway causes central apnea in awake and sleeping dogs. *Journal of Applied Physiology*.

[85] Malhotra A, Trinder J, Fogel R (2004). Postural effects on pharyngeal protective reflex mechanisms. *Sleep*.

[86] Sauerland EK, Orr WC, Hairston LE (1981). EMG patterns of oropharyngeal muscles during respiration in wakefulness and sleep. *Electromyography and Clinical Neurophysiology*.

[87] Fogel RB, Trinder J, White DP (2005). The effect of sleep onset on upper airway muscle activity in patients with sleep apnoea versus controls. *Journal of Physiology*.

[88] Tangel DJ, Mezzanotte WS, Sandberg EJ, White DP (1992). Influences of NREM sleep on the activity of tonic vs. inspiratory phasic muscles in normal men. *Journal of Applied Physiology*.

[89] Wheatley JR, Tangel DJ, Mezzanotte WS, White DP (1993). Influence of sleep on response to negative airway pressure of tensor palatini muscle and retropalatal airway. *Journal of Applied Physiology*.

[90] Farkas T, Kis ZS, Toldi J, Wolff JR (1999). Activation of the primary motor cortex by somatosensory stimulation in adult rats is mediated mainly by associational connections from the somatosensory cortex. *Neuroscience*.

[91] Lee S, Carvell GE, Simons DJ (2008). Motor modulation of afferent somatosensory circuits. *Nature Neuroscience*.

[92] Sawczuk A, Mosier KM (2001). Neural control of tongue movement with respect to respiration and swallowing. *Critical Reviews in Oral Biology and Medicine*.

[93] Sciortino KF, Liss JM, Case JL, Gerritsen KGM, Katz RC (2003). Effects of mechanical, cold, gustatory, and combined stimulation to the human anterior faucial pillars. *Dysphagia*.

[94] Yahagi R, Okuda-Akabane K, Fukami H, Matsumoto N, Kitada Y (2008). Facilitation of voluntary swallowing by chemical stimulation of the posterior tongue and pharyngeal region in humans. *Neuroscience Letters*.

[95] Kajii Y, Shingai T, Kitagawa J-I (2002). Sour taste stimulation facilitates reflex swallowing from the pharynx and larynx in the rat. *Physiology and Behavior*.

[96] Chen JC, Liang CC, Shaw FUZ (2005). Facilitation of sensory and motor recovery by thermal intervention for the hemiplegic upper limb in acute stroke patients: a single-blind randomized clinical trial. *Stroke*.

[97] Chen JC, Shaw FUZ (2006). Recent progress in physical therapy of the upper-limb rehabilitation after stroke: emphasis on thermal intervention. *Journal of Cardiovascular Nursing*.

[98] Lim KB, Lee HJ, Lim SS, Choi YI (2009). Neuromuscular electrical and thermal-tactile stimulation for dysphagia caused by stroke: a randomized controlled trial. *Journal of Rehabilitation Medicine*.

[99] Rosenbek JC, Roecker EB, Wood JL, Robbins J (1996). Thermal application reduces the duration of stage transition in dysphagia after stroke. *Dysphagia*.

[100] Rosenbek JC, Robbins J, Willford WO (1998). Comparing treatment intensities of tactile-thermal application. *Dysphagia*.

[101] Rowe LD, Miller AJ, Chierici G, Clendenning D (1984). Adaptation in the function of pharyngeal constrictor muscles. *Otolaryngology—Head and Neck Surgery*.

[102] Sumi T (1969). Some properties of cortically-evoked swallowing and chewing in rabbits. *Brain Research*.

[103] Theurer JA, Bihari F, Barr AM, Martin RE (2005). Oropharyngeal stimulation with air-pulse trains increases swallowing frequency in healthy adults. *Dysphagia*.

[104] Lowell SY, Poletto CJ, Knorr-Chung BR, Reynolds RC, Simonyan K, Ludlow CL (2008). Sensory stimulation activates both motor and sensory components of the swallowing system. *NeuroImage*.

[105] Sörös P, Lalone E, Smith R (2008). Functional MRI of oropharyngeal air-pulse stimulation. *Neuroscience*.

[106] Hamdy S, Rothwell JC, Aziz Q, Singh KD, Thompson DG (1998). Long-term reorganization of human motor cortex driven by short-term sensory stimulation. *Nature Neuroscience*.

[107] Fraser C, Rothwell J, Power M, Hobson A, Thompson D, Hamdy S (2003). Differential changes in human pharyngoesophageal motor excitability induced by swallowing, pharyngeal stimulation, and anesthesia. *American Journal of Physiology*.

[108] Gow D, Hobson AR, Furlong P, Hamdy S (2004). Characterising the central mechanisms of sensory modulation in human swallowing motor cortex. *Clinical Neurophysiology*.

[109] Schwaber JS, Kapp BS, Higgins GA, Rapp PR (1982). Amygdaloid and basal forebrain direct connections with the nucleus of the solitary tract and the dorsal motor nucleus. *Journal of Neuroscience*.

[110] Garcia-Diaz DE, Jimenez-Montufar LL, Guevara-Aguilar R, Wayner MJ, Armstrong DL (1988). Olfactory and visceral projections to the nucleus of the solitary tract. *Physiology and Behavior*.

[111] Guevara-Aguilar R, Jimenez-Montufar LL, Garcia-Diaz DE, Wayner MJ, Armstrong DL (1988). Olfactory and visceral projections to the paraventricular nucleus. *Brain Research Bulletin*.

[112] Guevara-Guzman R, Garcia-Diaz DE, Solano-Flores LP, Wayner MJ, Armstrong DL (1991). Role of the paraventricular nucleus in the projection from the nucleus of the solitary tract to the olfactory bulb. *Brain Research Bulletin*.

[113] King BM (2006). Amygdaloid lesion-induced obesity: relation to sexual behavior, olfaction, and the ventromedial hypothalamus. *American Journal of Physiology*.

[114] Richardson BE, Vander Woude EA, Sudan R, Thompson JS, Leopold DA (2004). Altered olfactory acuity in the morbidly obese. *Obesity Surgery*.

[115] Murphy C, Schubert CR, Cruickshanks KJ, Klein BEK, Klein R, Nondahl DM (2002). Prevalence of olfactory impairment in older adults. *Journal of the American Medical Association*.

[116] Cerf-Ducastel B, Murphy C (2003). FMRI brain activation in response to odors is reduced in primary olfactory areas of elderly subjects. *Brain Research*.

[117] Patel RC, Larson J (2009). Impaired olfactory discrimination learning and decreased olfactory sensitivity in aged C57Bl/6 mice. *Neurobiology of Aging*.

[118] Martin B, Maudsley S, White CM, Egan JM (2009). Hormones in the naso-oropharynx: endocrine modulation of taste and smell. *Trends in Endocrinology and Metabolism*.

[119] Knafo S, Barkai E, Herrero AI, Libersat F, Sandi C, Venero C (2005). Olfactory learning-related NCAM expression is state, time, and location specific and is correlated with individual learning capabilities. *Hippocampus*.

[120] Jean A (1984). Brainstem organization of the swallowing network. *Brain, Behavior and Evolution*.

[121] Beckstead RM, Norgren R (1979). An autoradiographic examination of the central distribution of the trigeminal, facial, glossopharyngeal, and vagal nerves in the monkey. *Journal of Comparative Neurology*.

[122] Panneton WM (1991). Primary afferent projections from the upper respiratory tract in the muskrat. *Journal of Comparative Neurology*.

[123] Hanamori T, Kunitake T, Kato K, Kannan H (1998). Responses of neurons in the insular cortex to gustatory, visceral, and nociceptive stimuli in rats. *Journal of Neurophysiology*.

[124] Aldes LD, Boone TB (1985). Organization of projections from the principal sensory trigeminal nucleus to the hypoglossal nucleus in the rat: an experimental light and electron microscopic study with axonal tracer techniques. *Experimental Brain Research*.

[125] Luo P, Dessem D, Zhang J (2001). Axonal projections and synapses from the supratrigeminal region to hypoglossal motoneurons in the rat. *Brain Research*.

[126] Pinganaud G, Bernat I, Buisseret P, Buisseret-Delmas C (1999). Trigeminal projections to hypoglossal and facial motor nuclei in the rat. *Journal of Comparative Neurology*.

[127] Tsuboi A, Kolta A, Chen CC, Lund JP (2003). Neurons of the trigeminal main sensory nucleus participate in the generation of rhythmic motor patterns. *European Journal of Neuroscience*.

[128] Borke RC, Nau ME, Ringler RL (1983). Brain stem afferents of hypoglossal neurons in the rat. *Brain Research*.

[129] Strick PL, Preston JB (1978). Sorting of somatosensory afferent information in primate motor cortex. *Brain Research*.

[130] Chen JC, Liang CC, Shaw FUZ (2005). Facilitation of sensory and motor recovery by thermal intervention for the hemiplegic upper limb in acute stroke patients: a single-blind randomized clinical trial. *Stroke*.

[131] Sommerfeld DK, von Arbin MH (2004). The impact of somatosensory function on activity performance and length of hospital stay in geriatric patients with stroke. *Clinical Rehabilitation*.

[132] Patil SP, Schneider H, Schwartz AR, Smith PL (2007). Adult obstructive sleep apnea: pathophysiology and diagnosis. *Chest*.

